# Supplementary figures and images for: Modelling 3D saccade generation by feedforward optimal control
Source: PLoS Comput Biol. 2021 May 24;17(5):e1008975. doi: 10.1371/journal.pcbi.1008975 (PMC8177626; doi:10.1371/journal.pcbi.1008975)

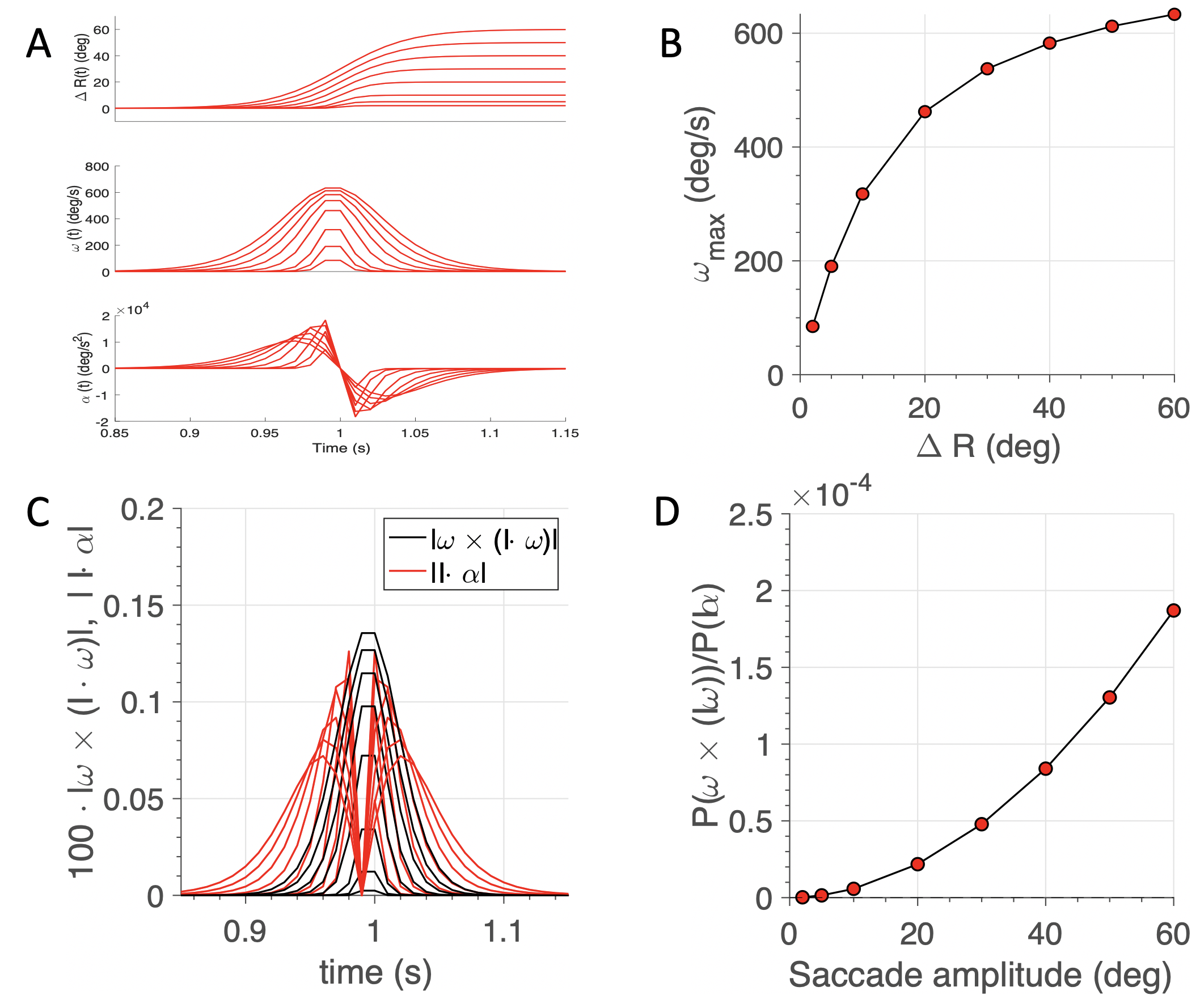

Supplement: S1 Fig — (A) Saccade-like traces (generated by an appropriately scaled tanh function): position (in deg) from 2 to 60 deg amplitude; angular velocity (in deg/s); angular acceleration (in deg/s2). (B) Main sequence of the simulated saccades. (C) Instantaneous value of the Iα and ω × (Iω) terms (taken from t = 0.85–1.15 s). Note differences in scale (factor 100) for the angular velocity (black) and angular acceleration terms. (D) The relative RMS power of the two terms (calculated over the same 300 ms as in C) as function of saccade amplitude. Its value increases with amplitude up to about 0.02% for the largest saccades. As a result, the latter term was ignored in our simulations. (TIF) [file pcbi.1008975.s001.tif]

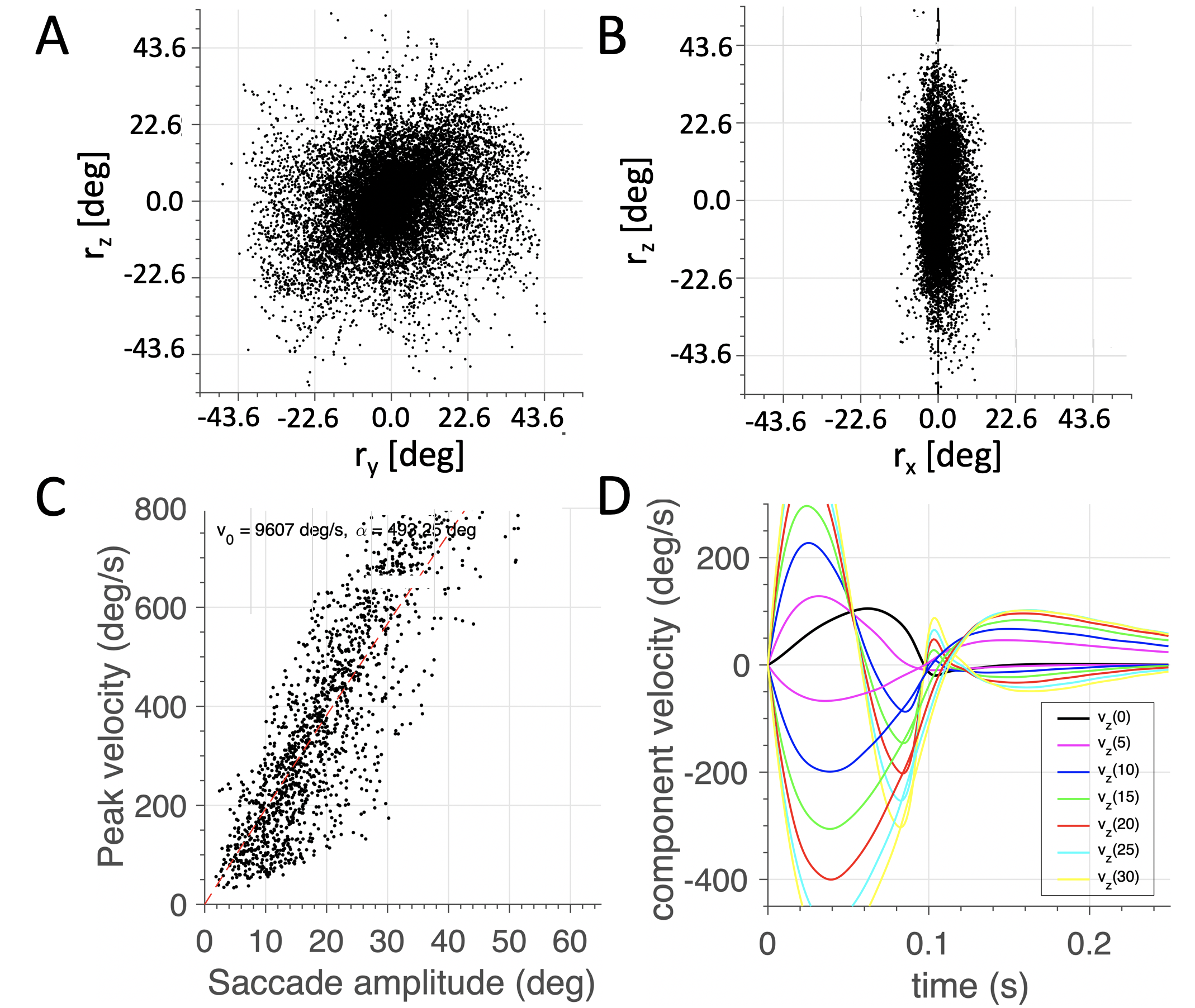

Supplement: S2 Fig — (A,B) 3D oculomotor behavior and (C,D) saccade dynamics, resulting from minimizing the AD cost. Although the 3D eye orientations are close to Listing’s law, the saccade dynamics (main sequence and component cross-coupling) are abnormal. The saccade peak velocity (C) doesn’t seem to saturate, as the exponential fit yields a non-physiological asymptote near 104 deg/s, with a angular constant close to the oculomotor range. (D) In the fixed-component oblique saccade test (as in Fig 9), the 8 deg horizontal component develops a large overshoot (with all durations the same, at about 100 ms), which is followed by a long return phase. This results in goal-directed, but curved saccade trajectories. See also Table 3 for numerical details. (TIF) [file pcbi.1008975.s002.tif]

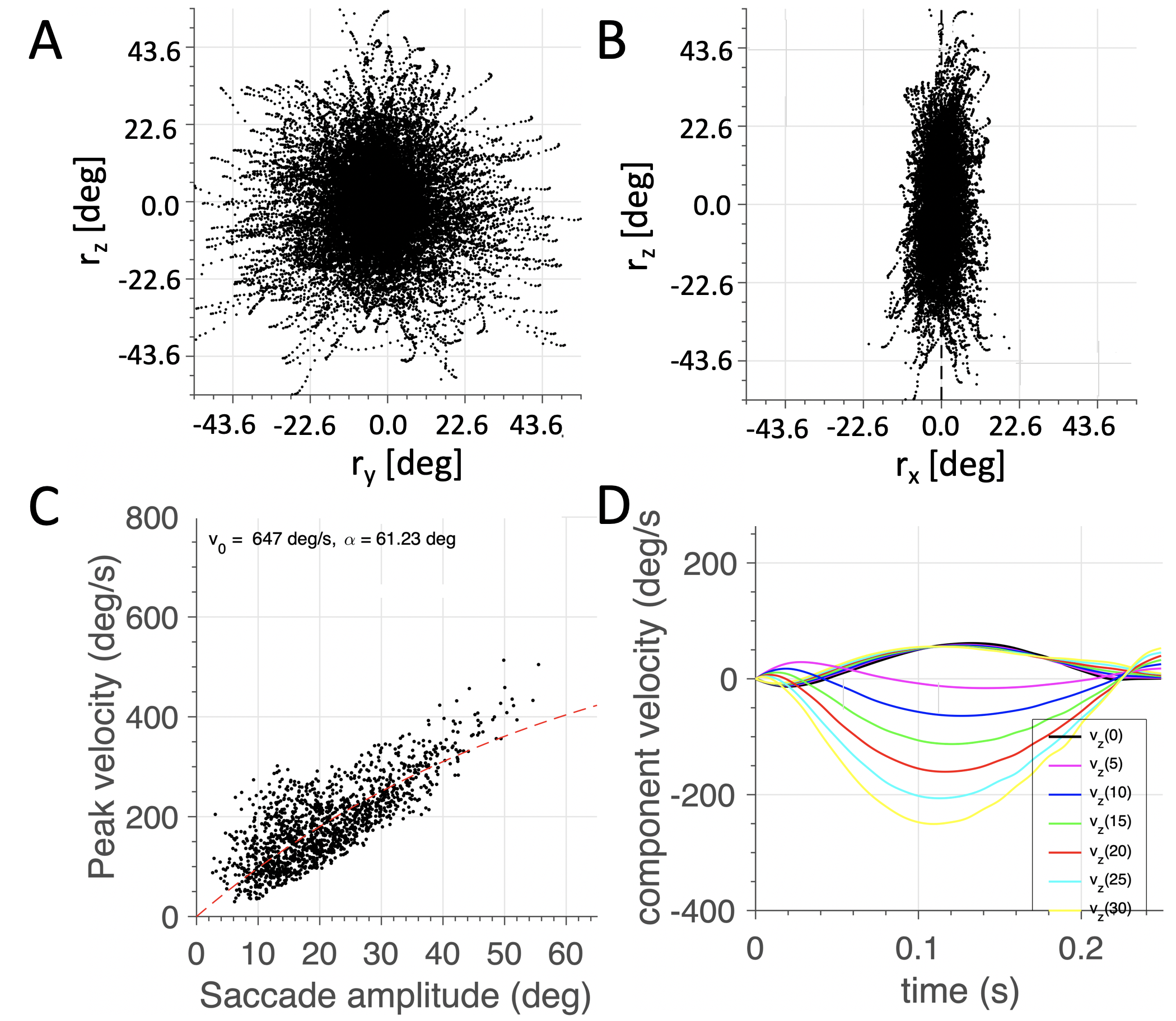

Supplement: S3 Fig — Also for this control strategy the saccades are abnormal: (C) the peak velocity increases linearly with saccade amplitude (but saccades remain much slower than for the JAD strategy, with the fitted asymptote at around 650 deg/s), and absence of component stretching (D), as all horizontal saccade components have the same, slow, velocity profile (all positive traces superimpose with a fixed duration of about 200 ms; See also Table 3 for numerical details). (TIF) [file pcbi.1008975.s003.tif]

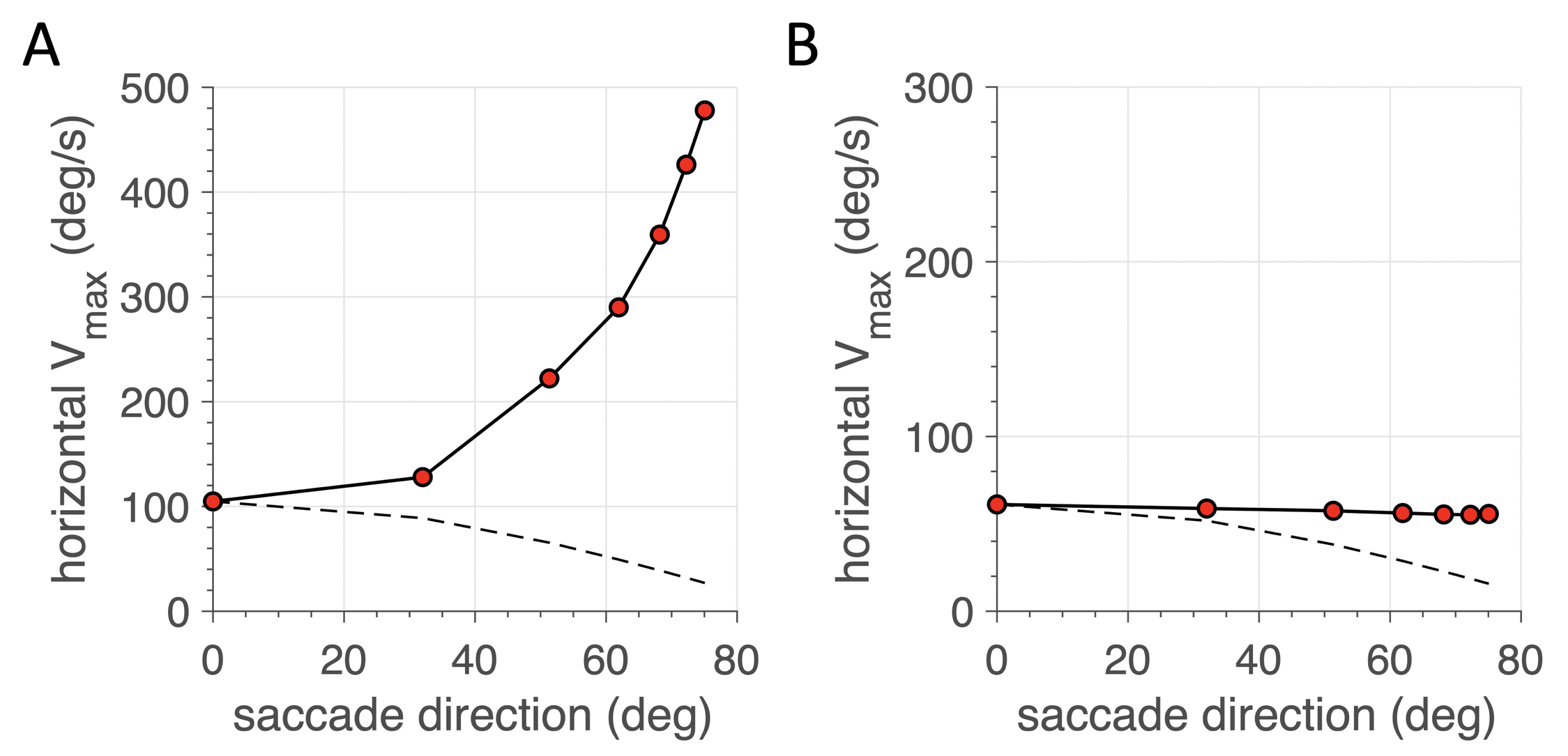

Supplement: S4 Fig — The simulated data have either a negative, or no correlation with the expected common-source cosine prediction (dashed lines; cf. Fig 11), indicating that saccade trajectories are curved. See also Table 3. (TIF) [file pcbi.1008975.s004.tif]

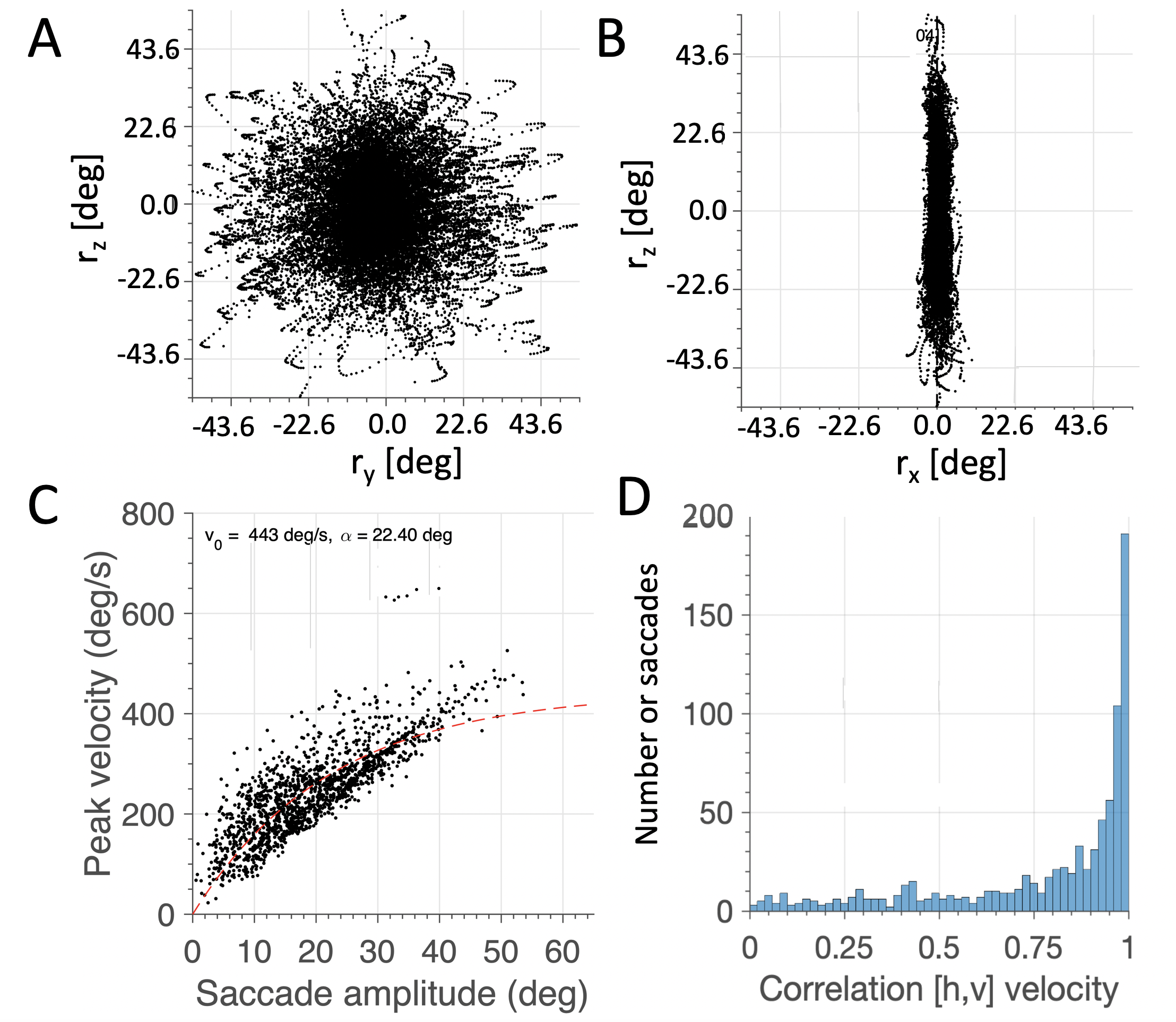

Supplement: S5 Fig — As expected, saccades follow Listing’s law (width of the plane is 2.25 deg), but peak velocities (C) of the saccades are much lower than for the strategies constraining AED and AED with final eye position in LP. (D) Saccade trajectories are straight, as the correlations between horizontal and vertical velocity profiles are high (see also Table 3 for numerical details). (TIF) [file pcbi.1008975.s005.tif]
