# Supplementary material for: Modelling 3D saccade generation by feedforward optimal control
Source: PLoS Comput Biol. 2021 May 24;17(5):e1008975. doi: 10.1371/journal.pcbi.1008975 (PMC8177626; doi:10.1371/journal.pcbi.1008975)
Supplement: Attachment — Submitted filename: Response to reviewers.pdf [file pcbi.1008975.s007.pdf]

Response to Reviewers

'Modelling 3D Saccade Generation by Feedforward Optimal Control'

Akhil John, Carlos Aleluia, A. John Van Opstal , Alexandre Bernardino

The text from the reviewers is transcripted in *italic* font and their questions numbered according to QX.Y (X is the number of the reviewer and Y is the sequential number of the question). The corresponding answers are identified by AX.Y, in normal font.

Reviewer #1: *While this is an interesting paper, I had to give it two full reads to understand where the authors were going with the paper.*

Q1.1. Ultimately, I reached the impression that it was written by two teams of authors, one with substantial physiological experience in the primate oculomotor system, and another with substantial experience in robotics and modeling. There is certainly nothing wrong with such a hybrid approach, but the paper seems more a concatenation of these two conceptual realms rather than a seamless integration. It is recommended that the authors revise the paper with attention to making it more readable, and one way to do that would be to better define the goal of the paper at the outset.

A1.1. The reviewer made a keen observation regarding our hybrid team. With the feedback and constructive criticisms from all three reviewers we have now substantially revised and considerably shortened our manuscript, and we have defined the goals of our paper early on. The mention of a simplified and upscaled robotic prototype and a clearer statement of the objectives are now presented in the Abstract and the initial paragraph of the Introduction. The figure with the illustration of our robotic prototype has been moved to an earlier section in the document, and is already referenced in the first sentence of the introduction.

Q1.2. Overall, the authors do support the case that penalties for accuracy, energy, and duration are insufficient to generate Listing's Law and hence commutativity of ocular rotations, but either the imposition of a penalty for the violation of Listing's Law at the saccade end point, or of total muscle force, give rise to reasonable Listing's type torsional behavior. This, of course, is applicable to the simplified robotic eye simulated here, but it would be nice if the authors made it clear from the outset that they are dealing with such a simplified robotic eye, rather than attempting to make it very representative of the actual biologic oculomotor system.

A1.2. We have now made clear from the outset (Abstract and first paragraph of the Introduction) that our purpose was to model a robotic eye that should generate the same oculomotor behavior as the primate eye on the basis of an optimal control strategy. Our robotic eye was not intended to faithfully mimic the detailed biomechanical properties of the human eye, so that we did not impose specific prior mechanical constraints on the ocular plant, like precisely tuned pulleys, which would by themselves already generate Listing’s law.

Q1.3. The omission of explicit pulleys, of course, substantially alters the properties of the oculomotor actuator, and thus would leave somewhat confusing the relationship between

Listing's Law as commanded entirely neurally, versus implemented to a large extent by the orbital connective tissue pulley system as appears to be the biologic case. Certainly, the controversy over this issue from about two decades ago was largely settled after the work of Ghasia et al. and Klier et al. from the Angelaki Laboratory. The actual biological system apparently does not implement Listing's Law by commands to the cyclovertical muscles for torsion, although certainly during eye-head coordination and vestibulo-ocular reflexes there exist violations. While this difference between the robotic eye modeled here and the biological one does not defeat the purpose of the paper, it would be worth making it clearer at the outset to the reader that there is a difference here that may be important.

A1.3. Indeed, the actual mechanical organisation of the eye plant will determine the neural commands needed to generate adequate saccadic eye movements that are accurate, straight, and comply with Listing's law. A specifically dedicated pulley mechanism, which tilts the ocular rotation axis exactly according to the half-angle rule would require driving signals encoding the coordinate velocity of the eye. However, any deviation from such a perfect mechanical solution would still require 3D ocular control signals, as has also been shown by human and monkey recordings.

Here we show that without imposing a particular muscle geometry with limited side-slip or pulleys for the horizontal muscles (note that our vertical and oblique muscles have been equipped with static pulleys) can generate LL without explicitly imposing it, basically by merely requiring two main optimization principles: (1) speed-accuracy trade-off, which ensures single-axis rotations of the eye-ball, nonlinear main-sequence kinematics in all directions and component cross-coupling, and (2) minimization of the total force exerted on the eye during fixations, which then ensures LL.

We do not entirely agree with the reviewer that the matter on LL has been 'settled' by the Klier and Angelaki results. Clearly, their finding that microstimulation of the abducens motoneurons did not violate LL for vertical initial positions, clearly points at a mechanical mechanism underlying LL, possibly instantiated by pulleys. It is important to note, however, that the induced movements were rather small (a few degrees) and slow (up to 50 deg/s), which is quite different from true saccadic pulse commands (velocities up to 1300 deg/s in the monkey). Further, for eye movements elicited in different directions for these same initial positions, the tilt of the angular velocity axis should have to be different (it varies systematically with the saccade angle), which has so far not been tested. Moreover, studies by Quaia and Optican (2011) have also indicated that muscle elasticity is highly nonlinear for increasing movement amplitudes and speeds, which will undoubtedly require a more sophisticated neural control for large and fast saccades than a simple linear velocity-to-position integration.

In addition, spontaneous saccades regularly violate LL (the law is not perfect, as illustrated in our Fig. 3C), and it has been shown that whenever a 'Listing error' becomes too large (exceeding about 1.0 deg), it is actively compensated by the next saccade, showing that saccades are, in fact, under 3D neural control, and not programmed as 2D displacement vectors.

It has been suggested that the systematic violations of LL during vestibular stimulation (Hepp et al., 1993) and eye-head movements (Tweed, 1998; Crawford and Vilis, 1991) might

be controlled by ‘active’ pulleys (Demer et al., 2000). However, Klier and Angelaki (J Neurosci 2012) demonstrated that during vertical/torsional vestibular stimulation, the plant mechanics did not account for the observed 3D oculomotor behavior. In other words, the brainstem circuitry accounts for 3D ocular control under all conditions; it simply has to account for the noncommutativity of sequential rotations (Tweed et al., 1999) but, clearly, it controls a 3D nonlinear plant with pulleys. We have now incorporated these arguments with the updated literature in our revised Introduction.

In summary, whether the plant necessitates a 3D rate-of-change of eye orientation (for a perfect pulley system, which fully implements LL), or angular velocity (no LL component at the level of the plant at all) has been settled in favour of the former for small and slow horizontal eye movements. However, it is quite conceivable that for saccades in all directions (not only involving the horizontal system) and at high saccade velocities, a pure ‘rate-of-orientation change’ signal will not suffice either, and that a significant contribution from neural encoding may be required too. In our paper, however, we do not elaborate on this interesting problem. Our simulations suggest that a relatively simple optimal control strategy could underlie the neural component to this problem.

These aspects are now more carefully explained in the document (see sections “3D kinematics” and “Straight Trajectories” in the Introduction). Additional explicit references to static pulleys in the vertical and oblique muscles of our robotic prototype were also inserted.

Q1.4. Figure 1, and throughout the paper. This reviewer finds it confusing and annoying that the authors seem to slip seamlessly between representation of angles as radians versus degrees, even within the same figure and its legend. It would be very helpful if the authors would consistently employ one system or the other, but not both. I prefer degrees, as does much of the published oculomotor physiology literature.

A1.4. We agree with the reviewer, and now resort to degrees throughout the paper. All figures (and figure captions) have therefore been updated.

Q1.5. Lines 42 through 46. Here the authors make some oblique references to the existence of the pulley system in the actual biological eye plant. Of course, there has been much development of the pulley idea since 1995, and the authors really should be citing the more recent references to the behavior of the peripheral oculomotor connective tissue apparatus and the muscle layers.

A1.5. In the Introduction, we have now included the papers from the Angelaki lab that discuss these ideas: Klier et al., 2006; and Ghasia and Angelaki, 2005, who show that there must be a pulley mechanism for the horizontal muscles that aid in the implementation of Listing’s Law. We also included the study of Tweed et al., Nature 1999, demonstrating the need for noncommutative neural controls, Tweed (1998), who showed the active control of ocular torsion during eye-head gaze shifts, and Quaia and Optican, 1998 (who worked out the mechanics of the pulley model).

Q1.6. Lines 48 and 49. The authors seem to be implying that Tenon described the pulleys in 2003, but in fact that is a reference to a translation of Tenon's work in 1816. Moreover, Tenon did not have much to say about the kinematic behavior that pulleys might have, so this

reference is not too helpful to the reader. Tenon's descriptions were barely even qualitative concepts of the anatomy.

A1.6. We agree. We have removed this reference from the list.

Q1.7. Page 3, lines 59 through 86. There is an extensive numerically labeled series of paragraphs about Listing's Law. While correct, much of this seems irrelevant to the message of the current paper, and probably should be deleted in the interest of brevity.

A1.7. We agree with this observation, and have now removed this extensive list of arguments. Instead, we stuck to the developments in the literature, described above.

Q1.8. Lines 90 through 95. Of course, the empirical observations cannot be accounted for by a passive pulley mechanism. Although the authors here mention the existence of active pulleys, it would be useful to say a bit more about their behavior, or else emphasize that the current model is not intended to have very much biological realism. Actual biological pulleys are active.

A1.8. We have now included a review of several papers that have discussed the possibility of active pulleys in the primate oculomotor system. Demer's paper (2000), who was the first to propose a role for active pulleys (that would allow for the vestibular violations of LL), the paper of Klier et al. (2012), which shows that the horizontal pulleys in fact are not active, and the paper of Misslisch and Tweed (2001), which provides theoretical arguments as to why an active pulley mechanism would require un-physiological movements of these pulleys during vestibular stimulation.

In our model we included static pulleys for the vertical/torsional muscles (but not for the lateral and medial rectus muscles); active pulleys were not considered in our simulations. A sentence clarifying this point has now been added to the relevant paragraph in the Introduction.

Q1.9. Lines 138 through 143. Here, the authors reveal clearly for the first time that they intend to analyze a simplified robotic eye rather than the actual biologic system. That is fine for theoretical purposes, but the robotic eye needs to be described in much greater detail than in Figure 3B. The model does not have any pulleys in the biological sense, and is insufficiently described.

A1.9. Indeed, we have split Fig. 3 (old versions) in two new separate figures (Fig. 1 and 2 - revised version), and improved their captions to better describe the role of the different muscles and characteristics of our robot eye. Our model actually includes pulleys for the vertical/torsional system, in order to ensure a large 3D oculomotor range, but we did not make any further claims about them in the original version of the paper. Now, we describe this aspect in the description of our robotic eye and make other explicit mentions to the pulleys in other relevant parts of the document.

The caption of the new Figure 2 now provides a more extensive description of our model.

Q1.10. Based on the use of centimeter dimensions later in the paper, one gets the impression that the authors may be talking about a large ophthalmotrope that might have

dimensions tenfold greater than that of the actual human eye and oculomotor system. The authors should greatly expand their description of the model eye and clarify that it is not intended to be particularly biological.

A1.10. Yes, this aspect has been clarified now since the beginning of the paper. The mention of a simplified and upscaled robotic prototype and a clearer statement of the objectives are now presented in the Abstract and the initial paragraph of the Introduction. The figure with the illustration of our robotic prototype has been moved to earlier in the document and is referenced in the first sentence of the introduction.

Q1.11. Line 163. The equation here including the imaginary numbers would seem to include some errors, because it is hard for this reviewer to understand how the product of ijk could be -1.

A1.11. In quaternion algebra, the imaginary numbers associated with the x, y, and z axes, here called i, j, and k, respectively, each obey i^2 = j^2 = k^2 = -1, but they are also mathematically related through the following cyclic relations:

i·j = k, and *k·i = j*, and *j·k = i*   Hence, from *i·j = k* it follows that *(i·j)·k = k^2 = -1*

It is through these latter cyclic properties that quaternions have a direct connection to rotations. The corresponding description in the paper has been complemented with the cyclic relations to make the algebra more clear.

Q1.12. Lines 190 and 191. This sentence does not on its face have much relevance to the paper and probably should be deleted as it is distracting.

A1.12. Indeed. The sentence was removed.

Q1.13. -Lines 210 through 221. Here the authors do explicitly disclose that their model does not replicate actual anatomic features of muscles. They also disclose that the way muscles change their direction of action and eye orientation changes is not physiologic either. That disclosure should be made earlier in the paper.

A1.13. We agree with the reviewer and we now disclose this in the abstract and introduction.

Q1.14. Table #1. The coordinates are specified in centimeters, rather than physiologic millimeters. This is okay for modeling purposes, but it took me as a reader some time to begin to understand that no attempt is being made here to make the model dimensionally realistic.

A1.14. The description of the model is now given much earlier in the paper, so we hope this perception no longer persists in the reader.

Q1.15. Lines 229 and 230, equation 18. It appears there may be an error in this equation, since the second equal sign probably is meant to be a plus sign.

A1.15. It is actually an identity sign. The total torque (term in the left), which is the sum of the elastic spring torques and the friction torque (term in the middle), is given by Euler's Equations for rigid body rotation (term in the right). We understand the formatting was

inadequate and ultimately led to the misunderstanding, so **the equation was reformatted** to make this “double” equality more explicit.

Q1.16. Line 241. Which term is the right-handed term of the equation that was neglected in the simulator? It would appear that there are multiple such terms, and with two equal signs in the equation it is hard for the reader to interpret what is stated.

A1.16. We have clarified in the text that it is the **cross-product term** that has been neglected.

Q.1.17. Line 270. How do the authors justify dynamic frictional force at this value? Assumed?

A1.17. Dynamical **frictional force was set** to make the robotic model system overdamped with similar time constants to the human eye. We did slightly rewrite the sentence where this was explained, to make it more explicit: “This parameter, which relates to the viscosity of the plant, was set to make the eye behave as an over-damped system”.

Q1.18. Line 295. Please define the acronym "PRBS."

A1.18. PRBS means **Pseudo-Random Binary Sequence** and is a signal often used in systems identification. It is a deterministic signal composed of rectangular pulses of fixed amplitude but variable duration that has similar properties to white noise (flat spectrum), thus excites equally all the frequencies of interest in the system. The acronym was expanded in text, and a footnote was added with the explanation above.

Q1.19. Page 18, Figure 8. Although in the text the meaning of the green dot is defined, it should be defined in the legend. In the lower right pane, the ordinate scale should be defined, although it is presumably the number of saccade events. It would be helpful to label the panels with the ABCD notation.

A1.19. The details of the dots have been included in the caption and the subfigures have labels now.

Q1.20. Figure 9. It would be helpful if the legend to this figure were a little bit more descriptive. It is not evident from the flow of the text what the message is or why this figure was included. That could be clarified.

A1.20. This figure shows samples of the trajectories of the saccades (same as in the previous figure), but now viewed projected on the x,z axes of a reference frame aligned with the best fit plane to the points (the Listing’s plane). This is implemented through a rotation explained in the text, whose equation is now included in the caption of the figure, to better explain the difference between this figure and the previous one.

Q1.21. Lines 491 and 492. The authors argue that the relative slowness of vertical saccades is because of mechanical actions between the obliques and vertical rectus muscles. This may be so, but it is not persuasive to merely state it without providing some evidence. It would be useful here and perhaps at other points in the paper to specify what the actual mechanical states are of the actuators.

A1.21. The main reason for this difference is that, while almost all the torque applied to the eye by the horizontal muscles is converted in horizontal motion, the vertical and oblique muscles divide their torques along the vertical and cyclo-torsional components (see Table 1). Furthermore, because of the pulleys, the force vectors applied to the eye by the vertical and oblique muscles are less orthogonal to the moment arm than the horizontal recti. Together, these factors reduce, in average, the effective torque, hence the peak velocity, in the vertical direction. This explanation is now inserted in the text.

Q1.22. Figure 14. The legends to most of the labels in the figure are too small to be legible. These appear to be smaller than 4-point font, and I could not read them at all when I printed out the paper.

A1.22. Legends have been removed from the figure. Corresponding values are reported in Table 3, so their presence as legends in the figure is superfluous.

Q1.23. Figure 15 legend. Once again, the centimeter units here are confusing, although I now suspect that the robotic model is much larger than the actual oculomotor apparatus that exists biologically.

A1.23. Same as A1.14.

Q1.24. Line 606. The paper correctly describes the model as having three independent motor signals for the three antagonist muscle pairs. It would be useful if this was more clearly stated at the beginning that this is not the way things work biologically, although that does not detract from the ultimate conclusions.

A1.24. We believe the extensions made in the abstract and introduction regarding the scope and objectives of the work, as well as the characteristics of the robotic prototype, now properly address this point.

Q1.25. Similarly, line 611 suggests that the muscles follow the shortest paths from their origins to insertions on the globe, a statement also inconsistent with what has been learned about the actual muscle paths through orbital imaging. Therefore, the conclusion in lines 613 through 615 that the model provides novel suggestions for potential principles underlying the neurobiological oculomotor system is rendered at least somewhat dubious. On lines 615 and 616, it is certainly plausible that this has direct applicability to robotic systems.

A1.25. We believe the changes in the manuscript now make clear we do not intend to model in detail the actual muscle paths. Anyway, we have rephrased these sentences to make more clear that the conclusions of the paper apply to simplified robotic models.

Old text:

"Our model provides novel suggestions for the potential control principles underlying the **neurobiological oculomotor system**, and it may serve as a starting point for more realistic biomimetic implementations in humanoid robotic systems."

New text:

“Our model provides novel suggestions for the potential control principles underlying the **emergence of Listing’s Law and the main-sequence in our robotic prototype**, and it may serve as a starting point for more realistic biomimetic implementations in humanoid robotic systems.”

Q1.26. Lines 636 and 637. This reader is still either unconvinced or does not understand the argument for the relative slowness of the vertical saccades. That idea deserves to be developed better.

A1.26. See our explanation to question A1.21. This has now been remedied also in the Discussion.

Q.1.27. Lines 654 and 655. It is an intriguing and important conclusion that force minimization can give rise to Listing's Law without other assumptions about the cyclotorsional state. That is a useful principle, although the behavior of the connective tissue apparatus and pulley system in the biological eye provides at least another basis for understanding the existence of Listing's Law.

A1.27. Yes, this is one of the main results of our study. We only claim that force minimization alone can explain Listing’s Law. We do not claim that the actual biological eye works this way.

Q1.28. Lines 754 through 760. It does not appear necessary for this paper to argue the alignment of vestibular canals in the extraocular muscles. This alignment is transient and approximate, and as the work of Ben Crane et al. has shown depends on the history of prior vestibular experience of the subject. This section should probably be deleted since the current model does not have any way of accounting for the biologic behavior.

A1.28. We agree; for the sake of length and focus, we therefore removed the sections “Vestibular Coordinates?” and “Neural Circuitry”, and moved a shortened version of “Consequences for humanoid robotics” to the introduction.

Q1.29. Lines 770 through 772. The authors should probably not stretch the point so much about the optimal control strategy and its resemblance to the human and oculomotor systems without providing data on the actual commands supplied to the muscles. In the absence of that comparison, the similarity is not convincing at the level of fine detail.

A1.29. Same as A1.28.

Q1.30. Supplemental Figure 1. It would be a favor to the reader to consistently use degrees or radians to represent angles. The reference for panels C and D talks about "the two terms," but leave the nature of these terms unclear.

A1.30. Figure and caption were changed accordingly.

Q1.31. Supplemental Figure 2. The legend to this figure is insufficiently detailed. Presumably, the authors expect the readers to compare this figure with some of the other named figures in the text, but the legend to this supplemental figure should really stand on its own.

A1.31. Same as A1.22.

Q1.32. Supplemental Figure 4. The symbols and dotted lines are insufficiently described. The legend to this figure should stand on its own.

A1.32. We have improved the caption of the figure.

Q1.33. Supplemental Figure 5. Once again, the legend is inadequate. The legend should stand on its own.

A1.33. We have improved the caption of the figure.

Reviewer #2: uploaded as an attachment (docx) and inserted inline here

Summary

In this manuscript, John et al. build a detailed mechanical model of the oculomotor plant and examine different emerging properties of movements produced by that model under various optimal feedback control (OFC) strategies. While it appears the primary goal of this work was to build a model capable of replicating known properties of saccade dynamics (e.g., its main sequence and Listing's law-adherent behavior), a secondary outcome of this work is to provide a theoretical basis that can help answer the age-old controversy of whether observed Listing's law are driven by muscolo-mechanical properties, driven by a neural control strategy, or possibly driven by both. The authors find that, using a model that includes some basic assumptions about the mechanical properties and geometries of the extra-ocular musculature together with a reasonable OFC cost function, normal saccadic kinematics, including the main sequence and Listing's law, can be replicated. This is an important and exciting finding for the oculomotor field, as, to my knowledge, this is the first time these two idiosyncrasies have been unified by a single model. The authors also describe final useful outcome of this manuscript is on the application-side: a working model of the oculomotor plant that generates physiologically-plausible 3D saccades. I find these contributions impactful, relevant and useful.

General comments

Q.2.1. In general, I thought this paper was very well-written, provides important findings, and is written with replicability in mind; however, I found it difficult to discern the primary rationale of the study until I was at least well-into the introduction. Indeed, the purpose/rationale is written in the Abstract "to explain [various saccadic oculomotor phenomena] we built a model [...]", but I feel like this work could benefit from a clear outline of the rationale at the start of the introduction section, before diving into the "Saccade 3D kinematics" details. This would help guide the reader a bit more explicitly on the rationale driving this work.

A.2.1 As explained above in our rebuttal to Reviewer #1, we have rewritten the Abstract and the Introduction, and now come to the main goals of our work from the outset.

Q.2.2 I find the Methods and Results sections to be well described, but I am left wanting more with respect to the Discussion section. For example, I think that it is necessary in any

computational work to devote a significant amount of the manuscript discussing the underlying assumptions made by the model and the limitations of its applicability (for example, this model ignores binocular 3D gaze orienting and therefore the extended Listing's law, L2). An explicit discussion of model assumptions and limitations seems to be missing.

A.2.2 We have now tried to make clear from the outset that the main purpose of our paper was to investigate whether simple optimization principles could underlie the observed nonlinear and cross-coupled dynamics and 3D kinematics of human saccades, without imposing explicit biomechanical constraints on the muscle geometry and their properties. In the Discussion we summarize the emerging properties of this simple model (nonlinear kinematics in all direction, straight trajectories and component cross-coupling, Listing's law), and also discuss the underlying assumptions in our model and their main limitations (e.g., biomechanical realism regarding the pulleys and actual muscle trajectories, and the nonlinear force-length relationships of muscle). The important result from our model is that the (limited set of) optimal control principles can generate all observed 3D oculomotor behaviors without having to impose additional biomechanical realism. We believe (and discuss, e.g. near Figure 17) that this framework can be extended to more complex behaviors and systems, like binocular control, nonlinear muscular properties, other geometries, and 3D eye-head coordination. Some of these will be topics for our future studies, but we feel that their detailed discussion here falls out of the scope of the present work.

Q.2.3 As well, I am left wondering about the generalizability/extension of their model framework to include other oculomotor mechanisms. For example, hypometric saccades are typically hypothesized to be an energy-preserving behavior to minimize backwards corrective saccades after an initial primary saccade - this model seems perfectly suited to studying this phenomenon, and I would be curious to read the authors' thoughts.

A.2.3 Indeed, the theoretical work of Harris (Vision Res, 1995; not included in our reference list, but his other work on optimization is included) had demonstrated that the typical 10% saccadic undershoot strategy could be due to a control that minimizes the total (statistically average) time of target acquisition in the presence of noisy signals (e.g. poor peripheral retinal resolution), in combination with the main sequence (larger saccades take longer), and increased cross-hemispheric signal transmission time in case of overshoots. We have not explicitly incorporated an additional cost in our model for an asymmetric signal transfer for left vs. right (as our model did not contain separate pathways for ipsilateral vs. contralateral movements), but in principle, adding such a cost (in software) would influence the control principles for saccade behavior. However, to incorporate such a strategy, one should also add a mechanism to reprogram and generate corrective saccades (requiring target remapping, as in double-steps); such a mechanism was not (yet) incorporated in our simple open-loop feed-forward model.

Q.2.4 Some other possible extension/generalization questions: while saccades are possibly the most frequent, smooth pursuit works synergistically with them to track moving objects in the field of vision (e.g., Orban de Xivry & Lefevre 2007).

A.2.4 We agree that in our future work other oculomotor behaviors, such as smooth pursuit, and eye-head-vestibular gaze control are interesting extensions of the model. However, as our paper was already quite lengthy, and had to be shortened substantially (according to reviewers #1 and #3), we have not included a detailed discussion on these other topics.

Q.2.5 How would the authors extend their saccade model to account for this other ecologically relevant scenario?

A.2.5 So far, we have not included task-constrained oculomotor, predictive, target remapping, and target-selection behaviors in our model. To include those in the model would require separate visual-spatial decision -, spatial remapping -, and selection mechanisms. The current simple eye model focused entirely on the motor control aspects of the oculomotor system, for which (for now) we assumed simple single visual target spots.

Q.2.6 What about fixational eye movements (e.g., Martinez-Conde et al. 2004)?

A.2.6 In our current model, small eye movements were part of the overall oculomotor repertoire. In other words, the saccades of our model formed a continuum from large to very small eye movements, in which microsaccades were not considered to be a separate class. The main reason for this was that the current eye-model was not (yet) driven by a true biomimetic visual input (as an inhomogeneous retina with a small high-resolution fovea). This will all change, though, once foveal visual input is incorporated in the model, and small eye movements will become crucial to scrutinize visual detail of targets on the fovea (in task-specific oculomotor behaviors). However, for now, this interesting extension of the model was not considered to be part of the study.

Q.2.7 What about the mechanism of saccadic adaptation (e.g., Pelisson et al. 2009),

A.2.7 The current model did not incorporate an adaptive mechanism that would respond to a consistent change in the stimulus-response relation for a particular target location as in the classical saccade-adaptation paradigm. Although we did not explore this in the present study, it would be conceivable to introduce a systematic stimulus-response mismatch after the model has been tuned. Like in the primate visuomotor system, an adaptive recalibration might then occur at the collective motor outputs. One would expect that this recalibration would be confined to a vectorial adaptation field around the adaptation target. However, like in A.2.3, the implementation of such a mechanism will require a target-updating mechanism, that so far has not been included in our model.

Q.2.8 or when the saccade target is less reliable (e.g., Lisi et al. 2019)?

A.2.8 This question addresses an interesting point to which we briefly allude in the discussion (section: Saccade dynamics). In the oculomotor system it has been found that central neurons are endowed with so-called multiplicative noise: the larger the driving signals, the larger their variability (e.g. the work of Harris and Wolpert). In optimal control models, like in ours, incorporating such noise sources has the same effect on the accuracy cost as the current energy term in our model (it introduces a quadratic term in the motor output; this has been shown, e.g. by Shadmehr and colleagues). In short, by including multiplicative noise (in combination with the accuracy cost) makes the energy cost in the control redundant, and in fact simplifies the optimal control: the J_AED cost simply becomes

J_AD. Moreover, multiplicative noise will also promote a more rapid acceleration phase of the saccades, and allow for a longer deceleration phase. In this way, it automatically accounts for positively skewed velocity profiles.

These are just a few topics I think readers from the oculomotor community might find interesting to discuss.

Detailed comments

Q.2.9 Lines 138-143: point of preference, but I think it would be clearer for the reader if a general description of this problem rationale and study purpose were presented at the start of the paper.

A.2.9 As explained in our response to the first reviewer, we have extensively rewritten our Introduction, and introduce our robotic system and goals at the outset.

Q.2.10 Lines 192-209: were there any probabilistic signals in this model? It seems the pseudo-random binary sequence may have done this, but I have a hard time interpreting this paragraph as a novice in (non)linear systems theory. At any rate, a clear description of signal variability may help for relating to the biological oculomotor system, as it is of course stochastic.

A.2.10 No, the current model is entirely deterministic. The PRBS signal is a deterministic signal with the same spectral properties as white noise (i.e., a flat spectrum) to be able to identify all aspects of the dynamical system. This has now been better explained in our Methods section.

Q.2.11 Line 410: 10 ms temporal resolution is very low res for saccade dynamics! Doesn't this influence (depress) the maximum peak velocities detectable during periods of high acceleration? Why did the authors set the time-step to this value?

A.2.11 Since the optimal duration of the saccade is not known a priori, and the search procedure was computationally quite intensive, we decided to calculate the duration costs for a range of durations in intervals of 10 ms to determine the optimal saccade duration. Thus optimal saccade durations were estimated at 10 ms resolution. However, the time resolution of the actual simulated saccades was 1 ms, and thus did not affect the velocity profiles of the saccades.

Q.2.12 Lines 474-479:

a. It is not readily apparent to me why well-correlated velocity components describe a straight trajectory.

A.2.12 This requirement of oblique saccades is described in the Introduction: when oblique saccade trajectories are exactly straight, the horizontal and vertical velocity components are (by definition) scaled versions of each other. In our data analysis we therefore defined a measure that readily describes to what extent the horizontal and vertical velocity profiles were scaled versions, which was their mutual correlation. If the correlation is 1.0 they are perfectly matched. In our simulations we obtained for most saccades correlations that were close to this optimal value.

Q.2.13 b. I'm actually quite surprised that some correlations were so low in the histogram. What does such a correlation imply for the trajectory shape? And how frequently did this happen? Perhaps showing some distribution quantiles relative to 1 (e.g., p95, p75, p50, p25, p05) would be helpful

A.2.13 As the 1500 test saccades were generated in random directions from random initial positions, it happened that sometimes they were close to the oculomotor range, and trajectories could become (quite) curved. We did not attempt, however, to precisely quantify the amount of curvature in each trajectory. For most of the optimization functionals in Table 3, however, very low correlations were exceptions. Deviations from the optimal value of 1.0, however, were common also because of the complex interactions of the six muscles in combination with the fact that the antagonistic muscle pairs were driven by independent motors. Such slightly curved trajectories are also observed for real human and monkey saccades. The real reason for this is unknown, but it is likely that the complex mechanics of the oculomotor plant plays a major role. However, strongly curved trajectories were most common for the J_AD and J_AE optimization cost functionals, as these functionals did not 'punish' deviations from single-axis rotations (that principle minimize either the total energy spent, or the saccade duration (shortest path)).

Instead of quantifying the relative number of curved saccades (which was approximately the same for all other cost functionals), and trying to quantify their 'curvedness', we took the prediction of the vectorial common-source model as a more insightful measure. This measure underlies the neurophysiological control property of vector-component cross-coupling in the system. As our model did not have an explicit cross-coupling of the three motors (they were controlled independently), the emergence of this cross-coupling (and producing relatively straight saccades) is an important emerging property from the model and its optimal control requirements.

For the J_AD and J_AE cost functionals the data do not follow the common source prediction (horizontal peak velocity varies as the cosine of the saccade vector direction) at all (as shown in Fig S4). The other cost functionals all led to nearly single-axis rotations, and therefore large amounts of cross-coupling, closely in line with the common-source prediction. In the right-most column of Table 3 this feature is quantified.

Q.2.14 c. What is the y-axis label for this histogram? # of saccades?

A.2.14 Yes, it is the number of saccades.

Q.2.15 Lines 517-520: is there a citation to support this claim (a Listing's plane width of 1.71 deg is within the expected Listing's law range?)

A.2.15 For human saccades, the thickness of Listing's plane is in this order of magnitude. We now refer to the Tweed and Vilis, 1988 paper to substantiate this.

Q.2.16 Lines 526-529: citations to support the claim that the saccadic system has an estimate of ocular torsion?

A.2.16 This claim follows from the paper of Van Opstal et al. 1996 (Journal of Neuroscience) and 1997 (book), in which it is shown that the saccadic system corrects deviations from

Listing’s plane with 3D saccades. This behavior can only be understood if the system has precise knowledge about this deviation, and hence of the actual torsional state of the eye.

Q.2.17 General comment for all figures with text overlays on scatter plots: The text is very hard to read. Suggest repositioning to eliminate overlap with datapoints.

A.2.17 All figures have been updated and are now better readable. We took out the text overlays in all panels, as the numbers are all included in Table 3.

Q.2.18. 8. Lines 603-627 “Discussion: Summary”: I think the authors have ‘buried the lede’ here a bit, as I’m a bit surprised they did not explicitly comment on the optimal control strategies that seemed to generate the most physiologically plausible movements- to me, this feels like a main contribution of this work worth mentioning explicitly here.

A.2.18 We have now revised this section by starting with a brief statement regarding the major findings that emerged from our study. The emerging properties of our model are described in more detail in the follow-up section.

Q.2.19 9. Lines 700-702: The evolution of an oculomotor primary position might also be a part of an adaptation to the natural statistics of the environment, as some degree of convergence is necessary for stereo-driven 3D perception (e.g., Sprague et al. 2015).

A.2.19 Indeed, the underlying reason as to why the eye would follow Listing’s law (and even take the trouble to implement part of this strategy at the ocular plant through a pulley mechanism) could, apart from an optimal motor-control principle (as explored here), also subserve certain visual-sensory functions, like binocular alignment for stereoscopic depth perception or line orientations. This idea was also forwarded by Hepp (1997), and in studies that measured 3D binocular control (Van Rijn et al., 1993; Minken et al., 1994), to show that the eye follows Donders’ law, rather than Listing’s law, for different eye orientations. We have now inserted a short paragraph on this idea in the Discussion, and also included the reference to Sprague’s work.

Q.2.20 Lines 853-855: Another potential application of this work: building humanoid-representative oculomotor robots may also be useful for ground-truth benchmarking of many eye tracking systems.

A.2.20 Thank you for the suggestion, which we now included in the text.

Reviewer #3: *The paper is about modelling eye movement dynamics using optimal control, specifically addressing the saccade generation. The paper reads like a very good survey of the field, yet perhaps misses out a few optimal control related papers that I am aware of.*

Q3.1 See for example:

[1] Kardamakis and Moschovakis, Optimal control of gaze shifts.

[2] Saglam, Glasauer and Lehmen, Vestibular and Cerebellar contribution to gaze optimality.

A.3.1 Indeed, these studies focused on optimal control in the saccadic system, but concentrated on one-dimensional control only. Moreover none of these studies focused on component cross-coupling, nonlinear kinematics, or Listing's law in relation to these optimal control principles. For completeness, we have now also included these two papers in our Introduction.

Q3.2 Using optimal control strategy on a model of the eye, the paper tries to explain Listing's Law, straightness of the rotation vector trajectory on the Listing's plane and a main-sequence non linearity that has to do with acceleration and deceleration of the eye as a function of the amplitude of the saccade.

I would have liked that the paper explained Fig. 3(B), the mechanical model of the eye better.

A3.2 In our response to reviewer #1 we explained how we have dealt with the improved description of our mechanical system, and how we updated the different figures.

Q3.3 Likewise, Fig. 4, could be explained better, as well. The line diagrams are really not clear. Is there a `easily understandable' connection between the model and the human eye muscles?

A.3.3 See A3.2

Overall the paper claims that the model explains various properties of the saccade by choosing various different type of optimality criterion. A slight tilt of the Listing's plane is connected to the static force on the eye.

Q3.4 The paper could be considerably shortened. The focus of the paper should be on the model, that explains various properties of a saccade generator and how the components of the optimality criterion is connected to these properties. Connecting to robotic or humanoid control was not necessary and is not done well. At the end, the paper reads like a long winded prose that does not seem to end.

A.3.4 We have now substantially shortened the Discussion by taking out the sections on Vestibular coordinates and Neural Circuitry, and we shortened and moved the section on Consequences for Robotics (in part) to the Introduction. Similarly, we have shortened the Introduction, and now address the main goals of our study already from the outset.
