# Supplementary material for: Modelling 3D saccade generation by feedforward optimal control
Source: PLoS Comput Biol. 2021 May 24;17(5):e1008975. doi: 10.1371/journal.pcbi.1008975 (PMC8177626; doi:10.1371/journal.pcbi.1008975)
Supplement: Attachment — Submitted filename: 2020-11-PLOS_CompBiol_review_OFCSaccades_John_etal.docx [file pcbi.1008975.s006.docx]

Review: Modelling 3D Saccade Generation by Feedforward Optimal Control

by John *et al.*

# Summary

In this manuscript, John et al. build a detailed mechanical model of the oculomotor plant and examine different emerging properties of movements produced by that model under various optimal feedback control (OFC) strategies. While it appears the primary goal of this work was to build a model capable of replicating known properties of saccade dynamics (e.g., its main sequence and Listing’s law-adherent behavior), a secondary outcome of this work is to provide a theoretical basis that can help answer the age-old controversy of whether observed Listing’s law are driven by muscolo-mechanical properties, driven by a neural control strategy, or possibly driven by both. The authors find that, using a model that includes some basic assumptions about the mechanical properties and geometries of the extra-ocular musculature together with a reasonable OFC cost function, normal saccadic kinematics, including the main sequence and Listing’s law, can be replicated. This is an important and exciting finding for the oculomotor field, as, to my knowledge, this is the first time these two idiosyncrasies have been unified by a single model. The authors also describe final useful outcome of this manuscript is on the application-side: a working model of the oculomotor plant that generates physiologically-plausible 3D saccades. I find these contributions impactful, relevant and useful.

# General comments

In general, I thought this paper was very well-written, provides important findings, and is written with replicability in mind; however, I found it difficult to discern the primary rationale of the study until I was at least well-into the introduction. Indeed, the purpose/rationale is written in the Abstract “to explain [various saccadic oculomotor phenomena] we built a model [...]”, but I feel like this work could benefit from a clear outline of the rationale at the start of the introduction section, before diving into the “Saccade 3D kinematics” details. This would help guide the reader a bit more explicitly on the rationale driving this work.

I find the Methods and Results sections to be well described, but I am left wanting more with respect to the Discussion section. For example, I think that it is necessary in any computational work to devote a significant amount of the manuscript discussing the underlying assumptions made by the model and the limitations of its applicability (for example, this model ignores binocular 3D gaze orienting and therefore the extended Listing’s law, L2). An explicit discussion of model assumptions and limitations seems to be missing.

As well, I am left wondering about the generalizability/extension of their model framework to include other oculomotor mechanisms. For example, hypometric saccades are typically hypothesized to be an energy-preserving behavior to minimize backwards corrective saccades after an initial primary saccade - this model seems perfectly suited to studying this phenomenon, and I would be curious to read the authors’ thoughts. Some other possible extension/generalization questions: while saccades are possibly the most frequent, smooth pursuit works synergistically with them to track moving objects in the field of vision (e.g., [Orban de Xivry & Lefevre 2007](https://physoc.onlinelibrary.wiley.com/doi/pdf/10.1113/jphysiol.2007.139881)). How would the authors extend their saccade model to account for this other ecologically relevant scenario? What about fixational eye movements (e.g., [Martinez-Conde et al. 2004](http://www.neuralcorrelate.com/smc/files/publications/martinez-conde_et_al_nrn04.pdf))? What about the mechanism of saccadic adaptation (e.g., [Pelisson et al. 2009](https://www.sciencedirect.com/science/article/abs/pii/S0149763409002073)), or when the saccade target is less reliable (e.g., [Lisi et al. 2019](https://www.pnas.org/content/pnas/116/32/16137.full.pdf))? These are just a few topics I think readers from the oculomotor community might find interesting to discuss.

# Detailed comments

1. Lines 138-143: point of preference, but I think it would be clearer for the reader if a general description of this problem rationale and study purpose were presented at the start of the paper.
2. Lines 192-209: were there any probabilistic signals in this model? It seems the pseudo-random binary sequence may have done this, but I have a hard time interpreting this paragraph as a novice in (non)linear systems theory. At any rate, a clear description of signal variability may help for relating to the biological oculomotor system, as it is of course stochastic.
3. Line 410: 10 ms temporal resolution is very low res for saccade dynamics! Doesn’t this influence (depress) the maximum peak velocities detectable during periods of high acceleration? Why did the authors set the time-step to this value?
4. Lines 474-479:
   a. It is not readily apparent to me why well-correlated velocity components describe a straight trajectory.
   b. I’m actually quite surprised that some correlations were so low in the histogram. What does such a correlation imply for the trajectory shape? And how frequently did this happen? Perhaps showing some distribution quantiles relative to 1 (e.g., p95, p75, p50, p25, p05) would be helpful
   c. What is the y-axis label for this histogram? # of saccades?
5. Lines 517-520: is there a citation to support this claim (a Listing’s plane width of 1.71 deg is within the expected Listing’s law range?)
6. Lines 526-529: citations to support the claim that the saccadic system has an estimate of ocular torsion?
7. General comment for all figures with text overlays on scatter plots: The text is very hard to read. Suggest repositioning to eliminate overlap with datapoints.
8. Lines 603-627 “Discussion: Summary”: I think the authors have ‘buried the lede’ here a bit, as I’m a bit surprised they did not explicitly comment on the optimal control strategies that seemed to generate the most physiologically plausible movements- to me, this feels like a main contribution of this work worth mentioning explicitly here.
9. Lines 700-702: The evolution of an oculomotor primary position might also be a part of an adaptation to the natural statistics of the environment, as some degree of convergence is necessary for stereo-driven 3D perception (e.g., [Sprague et al. 2015](https://advances.sciencemag.org/content/1/4/e1400254)).
10. Lines 853-855: Another potential application of this work: building humanoid-representative oculomotor robots may also be useful for ground-truth benchmarking of many eye tracking systems.
